# Regional Differences in Seasonal Timing of Rainfall Discriminate between Genetically Distinct East African Giraffe Taxa

**DOI:** 10.1371/journal.pone.0077191

**Published:** 2013-10-23

**Authors:** Henri A. Thomassen, Adam H. Freedman, David M. Brown, Wolfgang Buermann, David K. Jacobs

**Affiliations:** 1 Center for Tropical Research, University of California Los Angeles, Los Angeles, California, United States of America; 2 Department of Ecology and Evolutionary Biology, University of California Los Angeles, Los Angeles, California, United States of America; 3 Department of Atmospheric and Oceanic Sciences, University of California Los Angeles, Los Angeles, California, United States of America; University of Sydney, Australia

## Abstract

Masai (*Giraffa tippelskirchi*), Reticulated (*G. reticulata*) and Rothschild's (*G. camelopardalis*) giraffe lineages in East Africa are morphologically and genetically distinct, yet in Kenya their ranges abut. This raises the question of how divergence is maintained among populations of a large mammal capable of long-distance travel, and which readily hybridize in zoos. Here we test four hypotheses concerning the maintenance of the phylogeographic boundaries among the three taxa: 1) isolation-by-distance; 2) physical barriers to dispersal; 3) general habitat differences resulting in habitat segregation; or 4) regional differences in the seasonal timing of rainfall, and resultant timing of browse availability. We used satellite remotely sensed and climate data to characterize the environment at the locations of genotyped giraffes. Canonical variate analysis, random forest algorithms, and generalized dissimilarity modelling were employed in a landscape genetics framework to identify the predictor variables that best explained giraffes' genetic divergence. We found that regional differences in the timing of precipitation, and resulting green-up associated with the abundance of browse, effectively discriminate between taxa. Local habitat conditions, topographic and human-induced barriers, and geographic distance did not aid in discriminating among lineages. Our results suggest that selection associated with regional timing of events in the annual climatic cycle may help maintain genetic and phenotypic divergence in giraffes. We discuss potential mechanisms of maintaining divergence, and suggest that synchronization of reproduction with seasonal rainfall cycles that are geographically distinct may contribute to reproductive isolation. Coordination of weaning with green-up cycles could minimize the costs of lactation and predation on the young. Our findings are consistent with theory and empirical results demonstrating the efficacy of seasonal or phenologically dictated selection pressures in contributing to the reproductive isolation of parapatric populations.

## Introduction

Population divergence and speciation can result from genetic drift in geographic isolation, or from spatially variable natural selection [1], [2], even when gene flow is not completely restricted [3], [4]. Most studies of population differentiation focus on a single evolutionary mechanism, testing whether it has a significant effect on divergence. However, in order to assess the relative importance of neutral and adaptive processes, it is crucial to also consider the alternatives in a multi-model comparison. Here, we examine evolutionary processes that may maintain divergence in reproductively isolated East African giraffe taxa with abutting distributions. We will first introduce the problem of genetically distinct parapatric giraffe species, and then discuss four scenarios that might contribute to the maintenance of divergence, which we compared in this study.

Giraffes range from the Sahel to South Africa, living in scrub and savannah habitat in loose social groups with home range sizes between 5 and 992 km^2^ [5], [6]. They are highly mobile, capable of long-distance movements of 50–300 km [5]. Despite their mobility, giraffes are characterized by extreme genetic divergence amongst parapatric lineages [7]. Across Africa, at least six distinct groups can be identified, with little evidence of hybridization [7]. Recently, Groves and Grubb [8] treated these taxa as distinct species, and we will do the same in this paper. In addition, according to Groves and Grubb [8], there is little evidence to support a distinction between *Giraffa camelopardalis* and *Giraffa rothschildi*, and we will thus follow their suggestion by treating Rothschild's giraffe as *G. camelopardalis*. In East Africa, divergence between Masai (*G. tippelskirchi*), Reticulated (*G. reticulata*), and Rothschild's (*G. camelopardalis*) giraffe lineages is supported by strong genetic structure in mtDNA and microsatellites [7], and occurs despite the facts that these taxa have parapatric distributions [9], they are able to travel long distances [5], and they live in continuous acacia woodland habitat where barriers that could prevent movements among their respective ranges were seemingly absent in historic times, prior to anthropogenic habitat fragmentation [10]. Even though the species have different pelage patterns, with the potential for pre-mating isolation due to pelage-based mate recognition, individuals of these taxa hybridize readily in zoos [11], [12]. In contrast, cases of hybridization in the wild are rarely reported. The genetic evidence from mtDNA sequences indicates that the Masai giraffe has been separated from the Rothschild's and Reticulated giraffes since the early to middle Pleistocene (1.62 mya–0.54 mya) and the Rothschild's from the Reticulated giraffe since the middle Pleistocene (0.54 mya–0.18 mya), with minimal subsequent gene flow [7]. Consequently, it was suggested that the three giraffe taxa represent different species rather than subspecies [7]. In addition to clear genetic breaks between species, strong genetic subdivisions are also evident within species, particularly within the Masai giraffe [7].

Given the apparent absence of geographical barriers to dispersal, the striking genetic differentiation among these giraffes suggests that environmental or behavioural mechanisms limit gene flow. Here we consider four scenarios for the maintenance of divergence among the East African giraffe taxa, focusing on geographic and environmental parameters in a first-order assessment of their relative importance in discriminating between the three taxa. We make no claims concerning the environmental, orographic, or other conditions that initiated divergence among these taxa in the Pleistocene. Rather, we restrict our assessment to late Holocene to modern processes that contribute to the maintenance of the current, nearly complete reproductive isolation among the giraffe lineages. To this end, we conduct multivariate and spatially non-explicit as well as spatially explicit analyses to evaluate four hypothesized isolating scenarios: 1) isolation-by-distance; 2) the presence of barriers to dispersal, limiting gene flow; 3) spatial habitat differences that do not represent differences in timing of the seasons; and 4) differences in the seasonal timing of precipitation in relation to green-up.

Hypothesis I – Isolation-by-distance is the effect of diminishing genetic relatedness with increasing distance, and could potentially be important when dispersal is limited relative to the overall size of the range. Even though isolation-by-distance appears to be an unlikely force maintaining divergence between parapatric taxa, simulations suggest that under some circumstances parapatric speciation is possible solely due to limited dispersal distances and the accumulation of genetic incompatibilities [13], [14].

Hypothesis II – Geographic barriers to dispersal – and as a result gene flow – between the three giraffe species are not obvious, but they have been implicated – most notably the Rift Valley – in the divergence of other large mammals, including wildebeest (*Connochaetes taurinus*) [15] and impala (*Aepyceros melampus*) [16]. Thus, even though the ranges of giraffe species abut, dispersal might be limited by the steep topographical gradients of the Rift Valley and other habitat discontinuities associated with steep terrain. Dispersal limitation might be particularly strong among populations in the periphery of their distributions, and be present under either current or paleo-climate conditions. Given the mobility of giraffes, isolation-by-distance and geographic barriers are not strong candidates for the maintenance of divergence between the three giraffe species. For the sake of completeness, and to avoid bias by *a priori* ruling out any potential evolutionary process, we have nevertheless included both hypotheses in our analyses.

Hypothesis III – A third mechanism that might maintain reproductive isolation is divergent natural selection. Adaptation to local environmental conditions is increasingly viewed as a significant contributor to speciation (e.g., [2]). Habitat differences may reduce the fitness of dispersing individuals adapted to the habitat of the source population, resulting in population divergence, and ultimately leading to and maintaining reproductive isolation. Such divergence, often referred to as ecological speciation, may occur even in the face of ongoing gene flow [3], [4], [13], [17], [18]. Our third hypothesis focuses on spatially divergent general habitat conditions, but not differences in the timing of seasonal events. The latter is the focus of hypothesis IV.

Hypothesis IV – Finally, divergent natural selection can involve differential timing of reproduction [19], [20]. Most known cases entailing temporal isolation are restricted to narrow biological interactions, such as evolutionary divergence through disparate timing of host plant phenology (e.g., [19]). It was previously hypothesized that temporally distinct regional rainfall cycles, which coincide with the availability of high-quality browse, impose divergent selection regimes on reproductive timing in giraffes [7]. The synchronization of weaning with the availability of fresh browse represents a possible means by which temporal reproductive isolation could be favoured. Such synchronization could benefit both offspring and mother by increasing growth rates, hastening weaning, limiting exposure of calves to predation, and offsetting the female's energy debt as a result of lactation.

**Figure 1:**
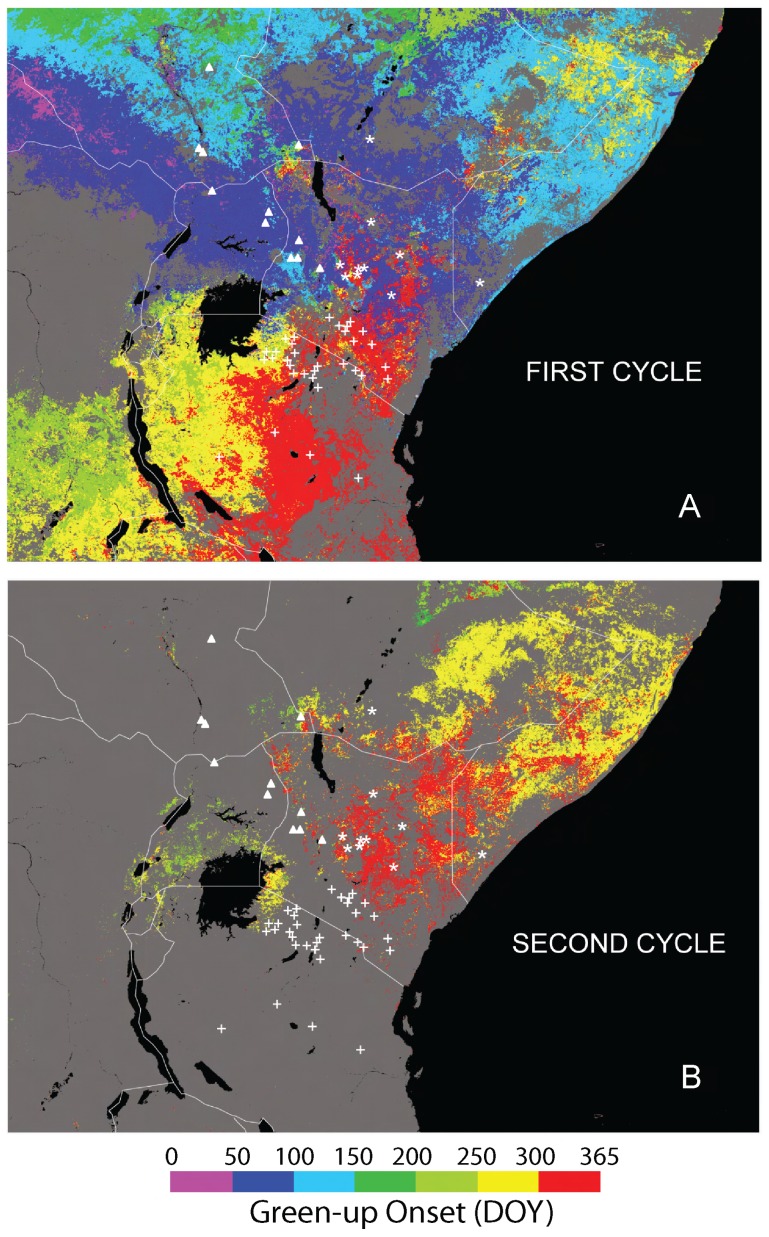
Spatial distribution of the day of the year (DOY) that green-up starts and giraffe point localities. Colors represent the day of the year that green-up starts. In some areas there are two seasonal cycles of rainfall and associated green-up. The start of the first cycle is shown in panel (A), and of the second cycle in panel (B). Point localities of genotyped giraffe samples are plotted in triangles (Rothschild's), asterisks (Reticulated), and pluses (Masai).

In East Africa, three regionally distinct seasonal cycles of precipitation correlate with the timing of green-up [21] (Fig. 1), when fresh browse becomes available. Peaks in precipitation in this region follow the season(s) of maximal insolation, shifting latitudinally during the year with the intertropical convergence, and producing regionally distinct rainfall patterns [21], [22]: 1) north of the equator, from northwestern Kenya through Uganda, July and August are the wettest months following the northern hemisphere summer solstice; 2) south of the equator, from southwestern Kenya through Tanzania, the rainy season occurs during southern hemisphere summer (December-March); 3) eastern Kenya, Somalia and Ethiopia experience bimodal precipitation, with maxima in spring (April-May) and fall (October-November), following maximal equatorial solar heating during the equinoxes. These regions generally correspond with the ranges of Rothschild's, Masai, and Reticulated giraffes respectively (Fig. 1). The Rothschild's giraffe was historically found in Uganda and Western Kenya [12]. The range of the Masai giraffe extends north through the Serengeti Plains and Masai lands up into Kenya, east to Mount Kilimanjaro, south to the Rufizi River, and west to Lake Rukwa and Lake Tanganyika. Finally, Reticulated giraffes occur from the Loroghi Mountains, the Barta Steppes, and Lake Turkana in the west to the Webi Shelbi River and the mountains of Ethiopia in the north, the dry coastal regions of Somalia in the east, and the Tana River in the south.

We tested how well each of the above hypotheses distinguishes between the three giraffe taxa using both non-spatially explicit and spatially explicit approaches. Because the more traditional methods to investigate associations between group membership and explanatory variables are non-spatial in nature, we start by focusing on environmental differences and differences in the timing of the seasons in a non-spatial context. Subsequently, we use more complex models that can specifically take into account the spatial relationships of populations as well as population connectivity.

## Materials and Methods

### Environmental variables

To capture the spatial distribution of parameters that are potentially useful in describing the giraffes' local habitat conditions, including those that relate to vegetation phenology, vegetation density, surface moisture, and topography, we used WorldClim climate data [23] as well as a suite of optical and microwave remote sensing data and derived products (Table 1). WorldClim bioclimatic metrics (WorldClim version 1.4 [23]) are derived from monthly temperature and rainfall climatologies [24] and are commonly used in characterizing habitat. They included eleven temperature and eight precipitation metrics, expressing spatial variations in annual means, standard deviations and extreme or limiting climatic factors. We checked for covariance among variables in our study area, and only included those with Pearson's correlations smaller than 0.9, resulting in a set of nine climate variables that were used in subsequent analyses (Table 1). We used this relatively high cutoff in order not to a priori rule out potential small but significant additive effects of correlated variables. To study the effect of temporal differences in rainfall patterns in more detail, we used the monthly climatologies from the WorldClim database [23], and calculated monthly rainfall as percentages of total annual precipitation, which will be referred to by ‘monthly rainfall’ and the name of the month in the remainder of this paper.

**Table 1:** Overview of the predictor variables used in this study.

Data Record	Instrument	Variables derived	Ecological attributes
Leaf Area Index (LAI) ‡	Satellite-MODIS		Vegetation density; net primary productivity
		LAImax	Annual maximum
		LAImin	Annual minimum
		LAIrange	Annual range (LAImax – LAImin)
Percent Tree Cover §	Satellite-MODIS	Treecover	Forest cover
Scatterometer-Backscatter †	Satellite-QSCAT	QScatMean	Annual mean surface moisture
		QScatsd	Standard deviation of surface moisture within a year
DEM	SpaceShuttle-SRTM	SRTM	Elevation
		SRTMsd	Elevation standard deviation (ruggedness)
		cost distances (CD)*	Permeability of habitat matrix based on elevation and ruggedness of the terrain
WorldClim ¶	Station-network	Bio1	Annual mean temperature
		Bio2	Mean diurnal temperature range
		Bio4	Temperature seasonality (standard deviation)
		Bio5	Maximum temperature of warmest month
		Bio6	Minimum temperature of coldest month
		Bio12	Annual mean rainfall
		Bio15	Rainfall seasonality (coefficient of variation)
		Bio16	Rainfall of driest quarter
		Bio17	Rainfall of wettest quarter
		Jan-Dec	Monthly rainfall as percentage of yearly total
NDVI **	Satellite-AVHRR	NDVImean	Annual mean vegetation greenness
		NDVIgreen	Greenness during greenest season
		Green-up	Day of year green-up starts
Distance			Geographic distance among sampling sites
Human population density	LandScan Global Population Database	Cost distances (CD)*	Permeability of habitat matrix based on human disturbance

Data at native resolutions smaller or larger than 1km have been aggregated to 1km.

† QSCAT annual mean and standard deviation are based on monthly data from the year 2001 with complete data coverage.

‡ LAImax, LAImin, and LAIrange are derived from monthly mean values based on the first 5 year of MODIS data (2000–2004 [26]).

§ Percent Tree Cover is based on MODIS data from 2001 [25].

¶ WorldClim data are based on monthly climatologies from 1950–2000 [23].

* Cost distances are computed either as Leas-Cost-Paths [48] or resistance distances [49].

** See [21].

Based on Moderate Resolution Imaging Spectroradiometer (MODIS) measurements on board of NASA's TERRA and AQUA satellites, we used the vegetation continuous field (VCF) product as a measure of the percentage of tree canopy cover [25], the Global Land Cover Dynamics product for vegetation phenology [21] and the leaf area index (LAI) product for vegetation density [26]. The spatial resolutions of these products based on optical passive measurements are 1km for leaf area index and vegetation phenology and 500 m for tree cover. To facilitate analysis, we aggregated the 500m native tree cover data to 1 km. The phenology fields capture the dates of onsets of green-up and dormancy of vegetation growing season cycles, and the algorithm was provided with the MODIS-based 16-day enhanced vegetation index (EVI) time series of the year 2001 to extract the respective dates [21]. To reduce processing and computation time, only one year of MODIS data was used. As a result, the vegetation phenology product has a considerable number of missing data points due to residual cloud cover. LAI is defined as the one-sided green leaf area per unit ground area. We averaged monthly LAI fields (Version 4) from the years 2000 to 2004 in order to reduce effects of residual cloud contamination [26] along with any natural inter-annual variability present in the data. The climatological monthly LAI composites were then used to generate three metrics: *LAI annual maximum* (LAImax), *LAI annual minimum* (LAImin), and *LAI annual range* (difference of maximum and minimum; LAIrange). These LAI metrics provide spatial information on vegetation density.

In addition to these optical remote sensing products, we included microwave QSCAT data available in three-day composites at 2.25 km resolution [27]. Data of the year 2001 were used to create average monthly composites at 1 km resolution and then further processed to produce two metrics that included annual mean and standard deviation of radar backscatter at horizontal polarizations. The QSCAT radar measurements, at wavelengths of ∼2 cm, are sensitive to surface canopy roughness, surface canopy moisture, and other seasonal attributes, such as deciduousness of vegetation [28]. For low density vegetation cover, such as woodlands, shrublands, and grassland savannas, the radar backscatter increases with increasing vegetation biomass and surface moisture [29]. Finally, for topography we used the Shuttle Radar Topography Mission (SRTM; http://www2.jpl.nasa.gov/srtm/) digital elevation data, aggregated from the native 90 m resolution to 1 km (available from the WorldClim group [23] at http://www.worldclim.org).

### Giraffe genetic and locality data

Giraffes (n = 429) from 51 locations throughout the ranges of the three focal species (Fig. 1) were collected and genetically typed for 14 microsatellite loci for a previously published study [7]. Sample collection, DNA extraction, and microsatellite analyses are fully described in [7]. Briefly, DNA was extracted from skin biopsies for microsatellite typing on an ABI 377 or 3100 (Applied Biosystems, Inc; Foster City, CA, USA). Fragment lengths were scored using GeneScan and checked for errors using MICRO-CHECKER 2.2.3 [30] and MSA 4.0 [31]. Nei's D and Fst between sampling sites were computed in Genalex 6 [32]. Genetic clusters were identified using Nei's D in POPULATIONS 1.2.28 (http://bioinformatics.org/~typhon/populations) and Bayesian clustering in STRUCTURE [33].

### Canonical variate analysis

**Figure 2:**
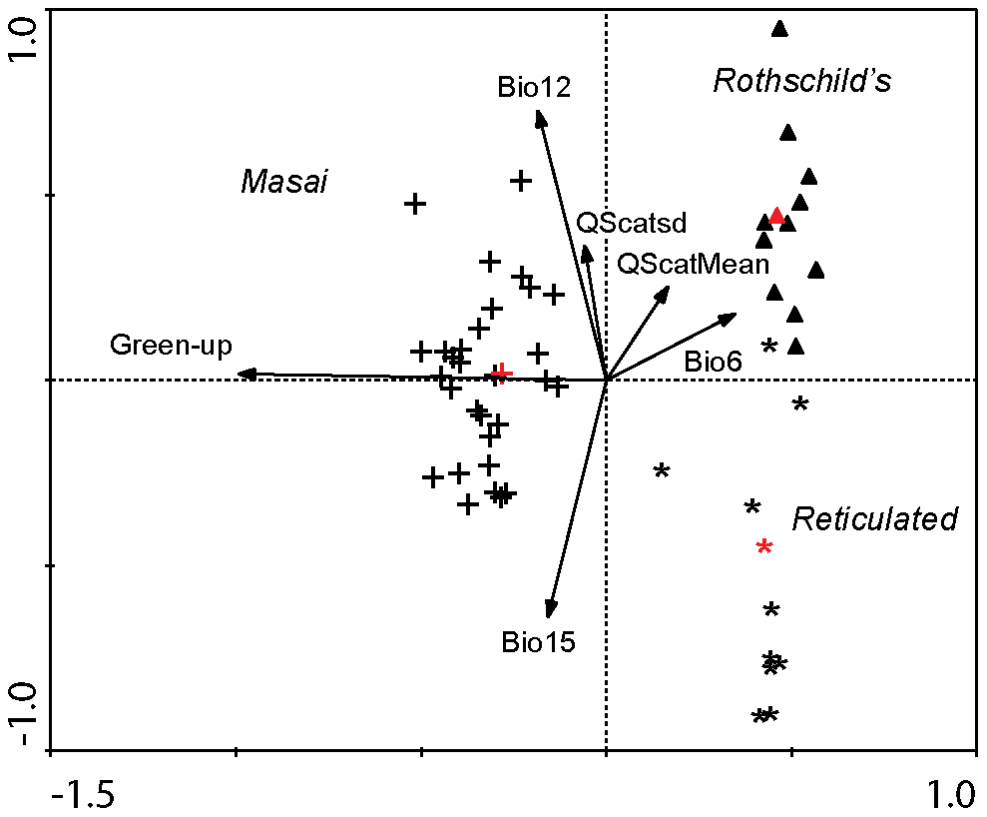
CVA ordination plot. Taxon centroids are in red; crosses  =  Masai; asterisks  =  Reticulated; triangles  =  Rothschild's; and vectors of environmental variables. Longer arrows indicate stronger contributions to the model, and their directions indicate degree of correlation with an axis. The first two axes explain 76.8% of taxon variation in environment. Bio6  =  minimum temperature of the coldest month; Bio12  =  annual precipitation; Bio15  =  rainfall seasonality (coefficient of variation); green-up  =  the day of the year of the onset of green-up; QScatMean  =  surface moisture (QSCAT); QScatsd  =  QSCAT standard deviation. See Table 1 and Methods for a full description of the environmental variables.

**Figure 3:**
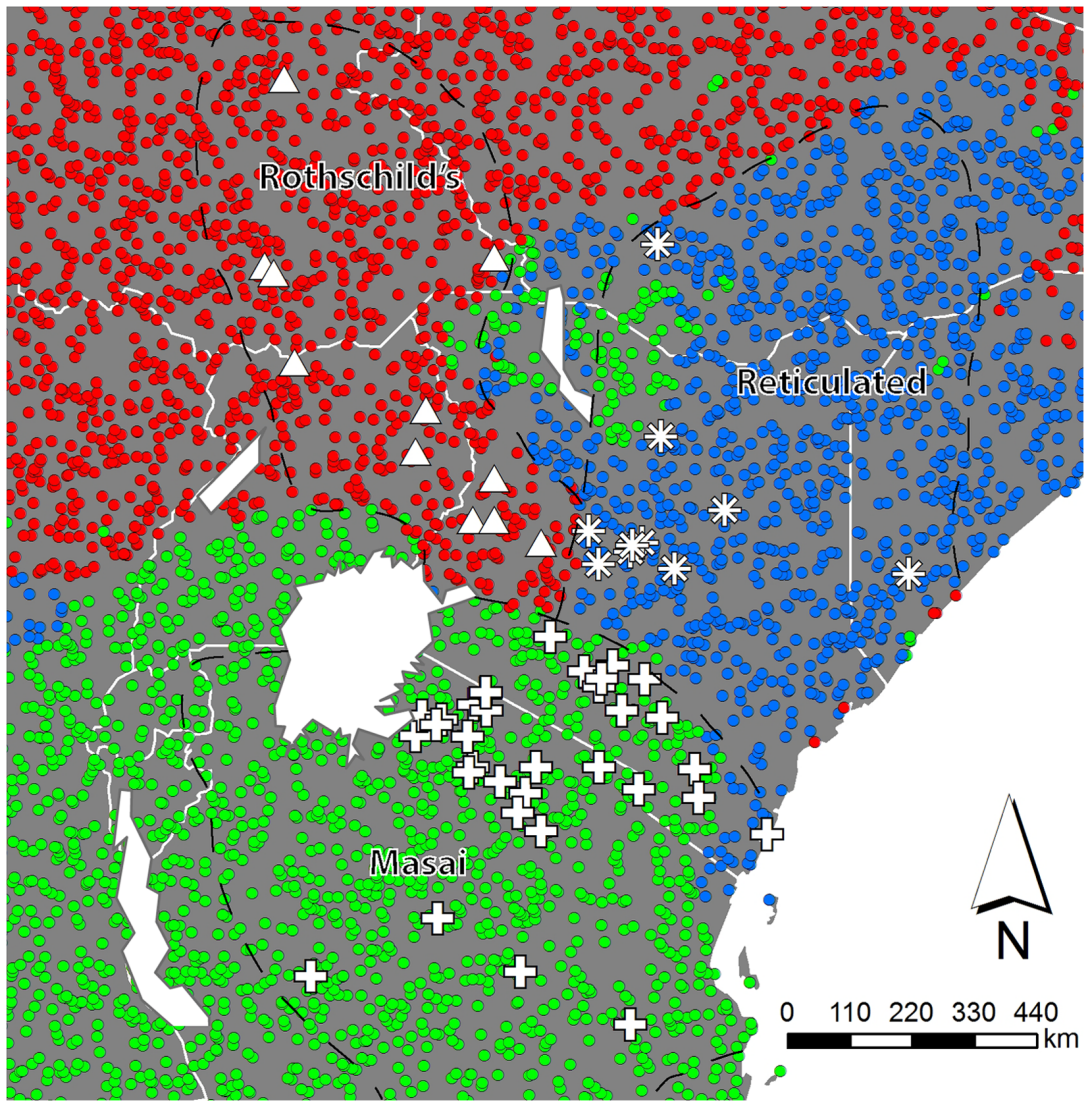
Results for random forest prediction. A random forest model based on taxon discrimination by monthly rainfall (Jan-Dec) was used to predict which taxon occurs at each of 10,000 randomly selected locations in the study area (coloured dots). Observed localities of the giraffe taxa are plotted in triangles (Rothschild's), asterisks (Reticulated), and pluses (Masai). Predicted taxon localities are indicated in red (Rothschild's), blue (Reticulated), and green (Masai). Approximate species ranges are indicated by dashed lines and their respective names (after [56]).

To test the hypothesis that the giraffe taxa occupy different habitats, a canonical variate analysis (CVA) was performed with CANOCO 4.5 [34]. We used genetic cluster membership (see also Figs. 2–3 for samples typed in [7]) to define the corresponding species at each sampling locality. For each species, a site was coded 0 if the species was absent and 1 if it was present. We then performed a canonical correspondence analysis (CCA), which is effectively a CVA with our data design [35]. A permutation test-based forward selection procedure was implemented to identify from the candidate set of environmental variables from WorldClim [23] and satellite remote sensing, those variables that best described habitat differences between the three taxa. We performed the forward selection procedure iteratively in order to exclude environmental variables that were highly correlated [36]. For a given iteration of the procedure, when a variable that was selected had a correlation coefficient r >0.75 with any of the previously entered variables, we excluded that variable, and re-ran the CVA. We re-ran the forward selection procedure in this manner until additional variables did not provide a significant improvement to the model. Bivariate correlations used to exclude variables were computed at 1000 random points throughout the study area. Significance tests on variables and on ordination axes employed 5,000 permutations each.

### Random forest models

To further test whether differences in the timing of rainfall could effectively differentiate among the three giraffe taxa as defined by genetic cluster membership, and to assess their importance relative to environmental variables that do not represent timing of seasons, we used random forest algorithms (randomForest v.4.5–30 [37]) as implemented in the R statistical framework (R Development Core 2009).

Classification tree models [38] implement binary recursive partitioning procedures to measure the amount of variation in a response explained by each predictor used in the model. No *a priori* assumptions are made about the relationship between predictor and response variables, allowing for the possibility of non-linear relationships with complex interactions. Homogeneity is measured by the Gini index [39], and splitting continues until further partitioning does not reduce the Gini index. Random forest methods incorporate a large number of these tree regressions, and for each tree constructed, use a random subset of the samples – the so-called bagging. Those samples not used in tree construction (the out-of-bag samples) are then tested against the random forest model, and error rates are computed across all runs to produce an estimate of classification error for the entire model [40]. Variable importance in random forest models is assessed by random sub-sampling of the variables and construction of new trees based on these predictor variable subsets.

First, we computed a random forest model using all predictor variables, including monthly rainfall (Jan-Dec) and bioclimatic and remote sensing variables. After verification that only monthly rainfall data were important contributors to the model, we computed a random forest model with only monthly rainfall variables, which was used in the subsequent predictive step. Random forest models were run with 20,000 trees (ntree  = 20,000), variable importance was computed (importance  =  TRUE), and default settings for the remaining parameters were used.

An imbalance in the number of records within a class (here, the number of sites where each species was identified) can bias random forest predictions, and cause high error rates in the classification of the rare class (e.g., [41], [42]). This phenomenon can be seen most frequently when imbalances of several orders of magnitude exist. Our dataset is imbalanced at a 1:1.1:3 (Reticulated: Rothschild's: Masai) ratio, but we nevertheless explored the iterative down-sampling approach developed by Evans and Cushman [41]. We generated 1000 random forest models with random subsamples of the largest class to a sample size of 10 and compared the average out-of-bag error rate and variable importance scores to the model run without subsampling.

Although random forests can be used with a large number of predictor variables, the out-of-bag error rate can be increased when many variables are included that do not contribute to explaining the response variable. To minimize the out-of-bag error rate while at the same time minimizing the number of predictor variables included, Murphy et al. [43] developed a new variable selection procedure that uses the variable importance scores (*I*) given as an output from random forests. For each variable *n*, its variable importance score *I_n_* is divided by the maximum variable importance score *I_max_*, resulting in a model improvement ratio (MIR) ranging between 0 and 1. In subsequent random forest models, variables with MIR smaller than a set threshold are withheld from the predictor variable set, and the resulting out-of-bag error rate is compared to that of the full model. We iteratively removed variables below MIR thresholds of 0-1at increments of 0.1.

Highly correlated predictor variables can potentially bias random forest results (e.g., [44]–[46]). To evaluate whether such a bias might be present in our results using the randomForest package, we confirmed variable importance using a conditional inference random forest algorithm implemented in the R package ‘party v. 1.0–6’. Conditional variable importance randomly shuffles the values of the predictor variable and computes a new model with the shuffled values. This new model is then compared to the one where the values were not shuffled. The difference in accuracy of the two models is indicative of the variable importance. Conditional random forest runs were run for 20,000 trees; remaining parameters were left at their default values.

To create a spatially explicit prediction of taxon distributions based on our random forest model, we extracted the values of the twelve monthly rainfall variables for 10,000 random points in a wide range of our study area. We used these points and our random forest model for only monthly rainfall data to predict (‘predict’ function in randomForest package) which taxon would be present at each of the 10,000 randomly drawn locations. The results were plotted on a map of the study area and compared visually to species distribution maps of the three taxa to evaluate the concordance between predicted and observed ranges. Because the available species distribution maps are rather crude approximations of the true ranges, we did not compute a percent overlap between predicted and observed ranges.

### Generalized dissimilarity models

*A priori* assignment of populations into one of the three study taxa would appear justified, because genetic evidence suggests clear divergence among those groups [7]. However, to further assess a potential bias of such an approach, and to explicitly assess the effects of isolation-by-distance or dispersal barriers as well as those of environmental heterogeneity, we also implemented a distance-based methodology using generalized dissimilarity modelling (GDM [47]). GDM is an expansion on matrix regression techniques to relate dissimilarities in predictor variables to dissimilarities in response variables, and make spatially explicit predictions of the predictor-response relationship into areas that have not been sampled. An advantage of GDM over other modelling methodologies is that it can explicitly take into account the influence of geographic distance and dispersal barriers on explaining biological variation, and allows for modelling variables that are difficult to define at individual sampling locations, such as genetic markers [47]. It can fit non-linear relationships of environmental variables to biological variation through the use of I-spline basis functions [47]. It is a two-step method: first, dissimilarities of a set of predictor variables are fitted to the genetic or phenotypic dissimilarities (the response variables). In an iterative process, predictor variables are added to and removed from the model, and only the variables that significantly improve the model are retained. Specifically, predictor variables are introduced to the model in random order and the variation in the response variable explained by the inclusion of that variable is compared to that without the variable (ΔD). Next, over many iterations the predictor variable is added again, but with the values randomized among sampling sites, resulting in a random distribution of ΔD_rand_. ΔD is compared to ΔD_rand_, based on which the predictor variable is either retained or dropped. Generalized dissimilarity models were run using an Avenue script in ArcView v 3.2 in conjunction with a SPlus v 4 script obtained from the authors of GDM [47].

To assess the level of population divergence, we used genetic distances (both Fst and Nei's D values) among the sixteen populations from our study taxa that were typed for fourteen microsatellite loci [7]. To characterize the regional timing of rainfall, we used the dissimilarity in monthly rainfall variables among sampling sites. In addition, to test for the influence of isolation-by-distance and dispersal barriers, we included geographic distance and least-cost-paths [48] or resistance distances [49]. Least-cost distances take into account spatial heterogeneity in permeability of habitats for dispersal. Least-cost-path and resistance distances were computed in Pathmatrix 1.1 [48] and Circuitscape 2.2 [49] respectively from friction surfaces that represented two types of barriers. First, giraffes generally do not occur higher than 2000 m above sea level [9], or in steep terrain. A friction surface representing potential ancient barriers was, therefore, based upon altitude and ruggedness (SRTM and SRTMsd respectively; see Table 1) of the terrain, which captured potential dispersal barriers formed by mountainous areas (e.g. regions in and along the Rift Valley). Values for SRTMsd ranged between ∼1 and ∼400, and were directly used as friction values in computations of cost distances. We similarly coded areas above 2000 m in altitude as 400, and those below as 1. Cost distances were then computed for altitude and ruggedness separately. We also added the values of the two friction surfaces for computation of a single cost distance matrix. Thus, areas above 2000 m in altitude, with the maximum level of ruggedness were ∼400 times as difficult for dispersal by giraffes as level areas below 2000 m. Because the assignment of costs is relatively arbitrary, we also computed cost surfaces for altitude and altitude+ruggedness where all values >1 (i.e. the minimum cost assigned to a grid cell) were divided by 10 and where those values were multiplied by 10. Thus, we computed the following cost distances: 1) ruggedness (untransformed cost surface); and 2) altitude and altitude+ruggedness for cost surfaces where the difference between the minimum and maximum cost was one, two, or three orders of magnitude.

The second friction surface represents more recent habitat changes by humans, and was computed directly from human population densities in East Africa (LandScan^™^ Global Population Database. Oak Ridge, TN: Oak Ridge National Laboratory. Available from http://www.ornl.gov/landscan). Although the relation between human population density and anthropogenic pressure varies from one region to the other (e.g., [50]), expanding human populations and increasing population densities may be proxies for land use changes (e.g., [51]) and other human-wildlife conflicts, such as cattle grazing (e.g., [52]), that affect the dispersal ability of giraffes. Because microsatellites evolve relatively rapidly, they may contain a signal of population divergence mediated by potential recent dispersal barriers resulting from anthropogenic land-use changes under the assumption of a generation time of approximately four years [7].

As a means to contrast the hypotheses regarding seasonal timing of rainfall, dispersal barriers, and geographic distance, we ran six models with different sets of predictor variables for both Fst and Nei's D. In two of these models all predictor variables were entered (full model), with cost distances based on either altitude or population density. Importance of any of those variables in a model would implicate its role in maintaining divergence among the three giraffe taxa. To evaluate cross-correlations among predictor variables, in the remaining four models the following subsets of the predictor variables were entered: only monthly precipitation variables, only geographic distance, or only one of the two cost distances. The percentages of the variation explained by each model were compared to assess which parameter set best explained the observed genetic variation.

### Frequency of giraffe births

To further investigate hypothesis IV (regional differences in the timing of rainfall), we assessed whether regional differences in the timing of green-up are related to reproductive timing in giraffes. Unfortunately, data on calving times in giraffes is largely lacking for our study taxa, and only available for two distinct genetic groups of Masai giraffe [7]: those in Nairobi National Park [53] and those in the Serengeti [54]. We first tested whether calving times conformed to a uniform distribution in each area, using Shapiro Wilks and Anderson-Darling tests. In addition, we tested whether the timing of births differs between these two regions using an autocorrelation analysis (acf function in the R Statistical Package), and compared this to the timing of maximum rainfall in each region.

## Results

### Tests of associations between predictor variables and giraffe divergence

We first assessed the roles of differences in the timing of green-up as well as general habitat differences in differentiating the three giraffe taxa in East Africa, by means of a canonical variate analysis (CVA) on a set of environmental variables pertaining to 51 locations where giraffe samples have been genetically typed (Fig. 1) [7]. The regional differences in rainfall maxima result in opposing seasons of green-up in the north and south of the region (Fig. 1a) and a second green-up in October/November in the north-eastern Kenya, eastern Ethiopia, and Somalia (Fig. 1b). Timing of first green-up, climate variables (Bio1-Bio17), and a number of satellite-derived ecological variables were included in the analysis (see Material and Methods and Table 2). The first and second CVA axes explained 47.4% and 29.4% of the variation among taxa respectively (F = 24.315, p = 0.0002). The first axis discriminated the Masai giraffe on the basis of first green-up (axis 1 vs. green-up, weighted r = −0.97; Table 3; Fig. 2). The second axis differentiated the Rothschild's and Reticulated giraffes, and was positively correlated with annual precipitation, and negatively correlated with Bio15 (precipitation seasonality not indicating timing, i.e. coefficient of variation; Table 3). This result strongly implicated the first green-up in differentiating the Masai giraffe. However, missing satellite data (Fig. 1) precluded analysis of the second/autumn green-up in much of Somalia, Ethiopia, and Kenya where we predicted it should differentiate the Reticulated giraffe.

**Table 2:** Overview of analyses conducted and hypotheses tested.

Analysis	Response variable	Predictor variables entered	Hypotheses tested
CVA	Taxon membership	LAI, Treecover, QSCAT, SRTM, Bio1-17, NDVI, Green-up	General habitat vs timing of green-up
RF	Taxon membership	Step 1: Same as CVA plus Jan-Dec Step 2: Jan-Dec	General habitat vs timing of rainfall Timing of rainfall
GDM F	Genetic distance §	Jan-Dec, cost distances*, distance	Timing of rainfall vs barriers vs distance
GDM E	Genetic distance §	Jan-Dec	Timing of rainfall
GDM D	Genetic distance §	Distance	Distance
GDM CD	Genetic distance §	Cost distances*	Barriers

§ Genetic distances were computed as Fst and Nei's D from microsatellite data.

* Cost distances include those based on elevation+ruggedness and human population density (see Table 1 and Material and Methods).

**Table 3:** Correlations between environmental variables used in the CVA analysis and the first two taxon ordination axes.

	Ax 1	Ax 2	Green-up	Bio6	Bio12	Bio15	QScatMean	QScatsd
Ax 1	1							
Ax 2	0	1						
Green-up	−0.97	0.01	1					
Bio6	0.34	0.14	−0.38	1				
Bio12	−0.18	0.56	0.17	−0.20	1			
Bio15	−0.15	−0.49	0.19	0.13	−0.50	1		
QScatMean	0.16	0.19	−0.16	−0.00	0.39	−0.04	1	
QScatsd	−0.06	0.28	0.13	0.14	0.09	0.20	−0.42	1

Bio6  =  minimum temperature of the coldest month; Bio12  =  annual precipitation; Bio15  =  rainfall seasonality (coefficient of variation); Green-up  =  day of the year of the onset of green-up; QScatMean  =  surface moisture (QSCAT); QScatsd  =  QSCAT standard deviation. See Table 1 and Materials and Methods for a full description of the environmental variables.

To extend the analysis to the second green-up, and to further investigate the timing component of the annual precipitation cycle that drives green-up in the three regions, we generated a monthly precipitation dataset, and used these variables (Jan-Dec) in addition to the satellite remote sensing and climate variables that capture general habitat characteristics to construct a random forest model [40], [55] (Table 1 and 2). Under this random forest model, most sampling localities were classified in their expected taxonomic group. The out-of-bag error rate was 3.8%, meaning that on average <1 locality showed a mismatch between observed and predicted taxonomic grouping. Out-of-bag error rate increased to 6.45% when we applied down-sampling of the largest class (Masai giraffe), where all Masai sites were correctly classified, and one Rothschild's and one Reticulated site were misclassified. However, after applying MIR to select the smallest set of variables that minimized the out-of-bag error rate, all sites were correctly classified (out-of-bag error rate  = 0%). The most important variables in explaining differentiation among taxa were qualitatively similar between runs where we did and did not apply down-sampling, and consisted of monthly rainfall in February and October, followed by March, August, July, and April (Table 4). These were also the variables retained after applying MIR. Moreover, the first five of those variables (February, October, March, August, July) were also the most important variables under the conditional inference variable importance criterion (Table 4). Rainfall measures in February and March, and in July and August are highly correlated (R^2^ <0.95), but this is of little to no influence on our random forest models. In each regression tree only one of the two correlated variables is picked as the most important variable. The presented importance scores are a summary of many tree regressions, and are an indication of how often each variable is used in a regression tree. Months known to be important in discriminating regional climate proved to be informative in the random forest model: February is associated with maximum precipitation in southern Kenya and Tanzania; July and August with maximum precipitation in north-western Kenya and Uganda; and March and October correspond to the post-equinoxal precipitation in north-eastern Kenya, Somalia, and eastern Ethiopia. Remote sensing and climate variables that do not capture the timing of seasons were relatively unimportant in the random forest model (Table 4), suggesting that general habitat differences alone cannot explain the observed taxonomic differentiation among giraffes in East Africa. A predictive map of the spatial distribution of our study taxa based on our random forest model corresponds with known taxon distributions [56] (Fig. 3). The one major inaccuracy is a prediction of Masai further north, in between Reticulated and Rothschild's predictions. This is an area in which many species distribution maps show a gap in giraffe occurrence (e.g., [56]). In an additional random forest model, we also considered the subdivision of Masai giraffe into two distinct units in the region, as suggested by molecular data [7], totalling four genetic entities in East Africa. The results of this model are comparable to that for the three giraffe taxa: an out-of-bag error rate of 3.7% and high importance of monthly rainfall variables, suggesting that seasonal timing of rainfall can also distinguish between smaller genetic entities.

**Table 4:** Random forest model results.

Predictor variable	Mean decrease in accuracy	Mean decrease in Gini index	Conditional variable importance
**Feb**	**16.14**	**4.2127**	0.0219*
**Oct**	**15.87**	**4.2656**	0.0719*
**Mar**	**14.58**	**3.4089**	0.0273*
**Aug**	**12.78**	**2.0622**	0.0422*
**Jul**	**12.43**	**2.0542**	0.0422*
**Apr**	**11.45**	**1.4400**	0.0124
Dec	11.07	1.6265	0.0129
Jan	11.02	1.6585	0.0182
Nov	9.19	0.9415	0.0056
Jun	8.42	0.9905	0.0209
LAIrange	8.13	1.0316	0.0143
Sep	8.03	0.8364	0.0084
NDVIgreen	7.13	0.7951	0.0158
Bio12	6.84	0.6681	0.0031
Bio16	6.18	0.6359	0.0048
NDVImean	5.44	0.5143	0.0051
Bio15	5.29	0.3559	0.0006
LAImax	4.91	0.4031	0.0065
Bio5	4.19	0.2783	0.0018
Bio4	2.26	0.0801	0.0000
May	1.74	0.1258	0.0000
Bio1	1.64	0.1204	0.0002
LAImin	1.39	0.1287	0.0065
Bio6	1.37	0.1285	0.0000
Bio17	0.89	0.0621	0.0000
QScatsd	0.82	0.1080	0.0001
QScatMean	0.42	0.0443	0.0000

Higher values of the “mean decrease in accuracy” and the “mean decrease in Gini index” indicate higher predictor variable importance. Variables in bold are the ones included in the random forest model that minimizes the number of variables used as well as the out-of-bag error rate after applying the model improvement ratio approach (see Material and Methods). Conditional inference variable importance is shown for a conditional inference random forest model, which corrects for potential biases due to correlations between predictor variables. Variables marked by ‘*’ are the five most important variables according to the conditional inference. The variables Jan-Dec represent the seasonal timing of rainfall; the remaining variables are representative of spatial differences in habitat. Also see Tables 1 and 2.

To explicitly assess the effects of isolation-by-distance or dispersal barriers as well as those of environmental heterogeneity, we also implemented a distance-based methodology using generalized dissimilarity modeling (GDM [47]). The full models for Fst values explained about 60% of the total observed variation, and those for Nei's D approximately 80% (Table 5). Monthly precipitation variables, in particular July and February, were consistently the most important in our models (Table 5; Fig. S1). The cost distances and geographic distance were also significant, but generally contributed far less to explaining the observed variation than precipitation variables. The only exception to this was observed in the model for Fst values with the cost distance based on human population density. Here, the cost distance was the second most important variable in the model, after July (Table 5; Fig. S1). In addition, models based only on geographic distance or the cost distances explained approximately 22%–78% less of the total genetic variation than the full models (Table 5). Whether the models with altitude or altitude+ruggedness cost distances were based on friction surfaces with one, two, or three orders of magnitude difference between low and high cost grid cells, made only negligible difference for the full models. However, for the cost-distance-only models (CD), the total variation explained ranged between 0.3% and 31.7%, the latter approximating that of the geographic-distance-only model (D) (Table 5). In comparison, models based only on precipitation variables performed nearly as well as the full models (Table 5). While rainfall values in some subsequent months are correlated, cross-correlated months are only included in the models if they have additive explanatory power. The interpretation of the selected months should, however, be general with respect to the timing of seasons, without assignment of any individual weight to cross-correlated months. The results from generalized dissimilarity models suggest that: 1) differences in timing of rainfall are important in discriminating among the three studied taxa; 2) dispersal barriers –in particular those imposed by human habitation- may have resulted in recent differentiation; and 3) isolation-by-distance played a relatively minor role in divergence among taxa.

**Table 5:** Generalized dissimilarity modelling results.

Genetic distance	Model	Cost distance entered in model	Percent of total variation explained (1/2/3 orders of magn.)*	Variables included in full model
Fst	F	alt+ruggedness	58.8/59.3/59.3	Jul, Feb, Jun, May, D, Oct, Sept, CD, Nov, Aug
		altitude	58.7/58.7/58.7	
		ruggedness	59.4	
		pop dens	60.9	Jul, CD, Feb, May, D, Aug, Oct, Nov
	E		58.6	
	D		21.3	
	CD	alt+ruggedness	9.9/2.5/0.8	
		altitude	22.7/22.7/22.1	
		ruggedness	3.0	
		pop dens	38.8	
Nei's D	F	alt+ruggedness	79.7/80.1/79.8	Jul, Feb, Oct, CD, Nov, May, D, Aug
		altitude	79.6/79.5/79.5	
		ruggedness	79.9	
		pop dens	79.9	Jul, Feb, Oct, CD, Aug, D, Nov, May
	E		79.3	
	D		31.2	
	CD	alt+ruggedness	13.4/2.1/0.3	
		altitude	31.7/30.1/30.0	
		ruggedness	2.7	
		pop dens	38.6	

Results shown are for six different models each on Fst and Nei's D genetic distances with monthly precipitation variables and geographic distance or cost-distances based on either altitude + ruggedness of the terrain or human population density. Variables entered in the six models were: full model (F: environment + distance + cost distance; E/D/CD); environment only (E); distance only (D); cost distance only (CD). Variables included in the models are only shown for the full models. CD =  cost distance; D =  geographic distance; E =  environmental variables (i.e. monthly precipitation); pop dens  =  human population density.

* For models where altitude and/or ruggedness of the terrain were entered, results are shown for cost distances based on one, two, or three orders of magnitude difference between suitable and unsuitable habitat. See Material and Methods for further details.

### Frequency of giraffe births

For differences in the timing of seasons to have biological meaning with respect to reproduction, calving times should also show differences between taxa. Tests of the null model of a uniform distribution of births across the year using the frequency of calving times from two distinct genetic groups within Masai giraffe [7] in Nairobi National Park [53] and the Serengeti [54] rejected a uniform distribution in both areas (Nairobi NP birth peak in August, September and one in January: Shapiro Wilks test, p = 0.0019; Anderson-Darling test, p = 0.0026; Serengeti birth peak in May-July: Shapiro Wilks test, p = 0.0205; Anderson-Darling test, p = 0.0191). In addition, an autocorrelation analysis (acf function in the R Statistical Package), testing the lag time where maximum correlation is observed between the frequency distribution of calving times in populations in Nairobi National Park and the Serengeti, showed that the highest correlation was observed at a lag time of 2–3 months, suggesting that these genetic units show differences in the timing of births. This corresponds to a similar lag time in peak rainfall and green-up in these areas.

## Discussion

We have investigated the potential current geographic and environmental factors that may contribute to maintaining divergence between giraffe taxa in East Africa. The results of our simultaneous tests of hypotheses presented here suggest that, among the factors investigated, regional differences in timing of maximum rainfall are of primary importance. Even though general habitat differences, dispersal barriers, and isolation-by-distance also appear to contribute to inter-taxon differentiation, our analyses suggest they play less important roles.

Previous studies have shown that parapatric speciation is possible when dispersal distances are limited and genetic incompatibilities accumulate, resulting in reproductive isolation of parapatric populations [13], [14]. Indeed, even though giraffes are able to travel large distances [5], dispersal may be limited due to small home range sizes and responses to limited resource availability [54]. Moreover, genetic structure is apparent not only between, but also within species [7]. Nevertheless, generalized dissimilarity models in which geographic distance was entered as a predictor variable suggested that it did not play a major role in explaining genetic variation in a spatial context (Table 5).

Studies using a resistance surface to compute cost distances are often subject to an oversimplification of the relation between the habitat matrix and gene flow, and our study is no exception. To obtain a better model of gene flow as a function of the habitat matrix, Shirk et al. [57] developed a novel approach in which the functional relationship between habitat characteristics and resistance are varied, resulting in a series of cost distances that are correlated to a measure of gene flow. For multiple habitat characteristics, this is first done in a univariate procedure. Subsequently, the univariate optimal functions are used to start a multivariate optimization procedure to find the resistance surface best describing the observed genetic divergence between populations. Even though the approach proposed by Shirk et al. [57] is a major improvement over approaches assigning arbitrary costs to certain habitat conditions, it depends heavily on detailed expert knowledge. First, the starting parameters in the optimization procedure are set based on expert knowledge, and second, the optimized parameter values should be evaluated to what extent they are biologically meaningful. Unfortunately, such expert knowledge is sparse for giraffes, which is why we chose not to follow the procedure outlined by Shirk et al. [57]. Our analyses of dispersal barriers resulting in a reduction of gene flow are, therefore, subject to limitations. Yet, our results are in line with those of Arctander et al. [15], who found low levels of divergence for several species across the Rift Valley, suggesting that the Rift Valley may not be a major topographical barrier to large mammals in general. It remains unclear to what extent human-induced barriers may influence divergence among giraffe populations and species. Generalized dissimilarity models of Fst values where human population density was included as a cost distance suggest that dispersal might be limited across areas with high human population densities. However, similar models for Nei's D were less conclusive in this respect. Given that human disturbance of the magnitude currently seen is a recent phenomenon, and giraffes have long generation times, the microsatellite data may not show a signature of reduced gene flow yet. Nevertheless, human occupancy does not appear to be related to habitat characteristics unfavourable to giraffe dispersal over longer evolutionary times.

Locally adaptive responses to divergent habitat could provide selective pressures favouring endemic populations over migrants or hybrids. The most significant habitat variables that are not related to the timing of the seasons in canonical variate analyses were Bio12 (annual precipitation) and Bio15 (coefficient of variation in annual rainfall). Rothschild's giraffe occupy habitat that generally gets more rainfall during the year than the regions occupied by Masai (medium levels of rainfall) and Reticulated giraffe (lowest levels of rainfall). Similarly, Rothschild's giraffe habitat is characterized by smaller seasonal differences in rainfall than that of Reticulated and Masai giraffe. The low levels of rainfall in Reticulated habitat result in the lowest observed vegetation cover during the greenest time of the year (NDVIgreen), as well as small differences in greenness between the seasons (LAIrange). Nevertheless, there is large overlap in these variables between the three giraffe species, and they are therefore not effective predictors of genetic divergence.

Although the association between patterns of green-up and giraffe taxa suggests a role for geographic differences in the seasonal timing of rainfall in the maintenance of differentiation, the historical processes of initial giraffe divergence remain an open question. The ∼ 23,000 year precession cycle and associated ∼ 100,000 year climate cycle strongly influence African precipitation and is known to have resulted in changes in precipitation intensity multiple times in the region since the early to middle Pleistocene [58]–[60]. This corresponds to the approximate time of the first split between these giraffe taxa, that of the Masai and Reticulated+Rothschild's giraffes [7]. Discrete rainfall regimes with comparable seasonal attributes likely persisted throughout the late Pleistocene and early Holocene, albeit with significant variation in precipitation intensity and geographic extents [61]. Spatially and temporally differentiated, these rainfall patterns could have contributed to initial divergence of the three giraffe taxa. Alternatively, as a result of the precession cycles, savannah habitat has repeatedly expanded and contracted [62], [63], a process that has been hypothesized to be the primary cause of divergence in several savannah mammals [9], [64]–[71]. Under the hypothesis of periodic isolation in savannah refugia, followed by range expansions tracking the expansion of savannah during more favourable arid periods, it is plausible that the three giraffe taxa in East Africa initially diverged in allopatry, and remained distinct through one of the mechanisms described above. Studies of paleoclimatic conditions and habitat suitability, as well as detailed genetic and demographic studies should provide insight into the causal mechanisms of initial divergence of giraffes as well as other African savannah species. Finally, even though least-cost-path analyses that consider orographic features suggest that they do not impose significant dispersal barriers causing reduced gene flow under current conditions, they may have contributed to initial giraffe divergence under historical conditions, when these features may have been more severe, or – combined with paleo-climate conditions – may have harboured less suitable habitat conditions.

The striking correlation between seasonal timing of rainfall and genetic divergence among giraffe taxa might be explained by different, but not necessarily mutually exclusive mechanisms. First, genetic divergence might be related to a synchronization of the reproductive cycle with regional timing of rainfall. For available data, a uniform distribution of births across the year was rejected, which is supported by independent data from the Serengeti [72]. This suggests that calving times in giraffes display seasonality. This is substantiated by observations of seasonal peaks in births among various giraffe taxa throughout Africa [73]–[78]. For East Africa, data is only available for Masai giraffe in the two above mentioned areas. These areas represent genetically distinct entities within the Masai giraffe [7] that also experience distinctly different rainfall regimes. Our finding that populations in Nairobi National Park and the Serengeti show differences in birth timing, is consistent with their unique signatures of seasonal timing of rainfall, the latter of which was confirmed by the random forest model on the four genetically distinct groups. Similar differences in seasonal timing of births between Masai, Reticulated, and Rothschild's giraffe seem to be supported: the preponderance of births appears to occur in the dry season (Fig. 2 in [54], [79]) (Fig. S2), which is January through March for Rothschild's [9] and May through August for Masai giraffes [54], [79].

Synchronization of the reproductive cycle with the timing of rainfall may involve selective advantages related to the condition of the female at the time of conception, increased growth rates and predation avoidance for calves, and quick recovery of the female at time of weaning. As a result of births peaking late in the dry season, weaning of giraffe young occurs at a time that fresh browse becomes available [80], [81]. This synchronization may be beneficial to both offspring and mother. First, consumption of high-quality browse by weaning calves during green-up may result in increased growth rates, hasten weaning and thus limit exposure of calves to predation, which is a major cause of mortality in wild giraffes [80]; lion and hyena predation on calves can approach 50% [54], [82]–[84]. Second, lactating females experience substantial costs minimizing predation risk [54], which could be offset by the abundance of browse. Females with young prefer open settings [85], sacrificing foraging opportunities for lowered predation risk to their calves [54]. This form of predation avoidance likely contributes to the maternal energy debt experienced by lactating females late in the dry season [86]. This energy debt does not occur in non-lactating adults [79]. Hastened weaning of offspring should reduce these energetic costs for females, and the availability of high-quality browse for females during and following the most demanding phase of reproduction could mitigate the impact of reduced foraging time. The importance of the availability of high-quality browse is consistent with observations that lactating females show stronger preferences for tannin-free, high-protein-level browse associated with green-up than adult males or non-lactating females [72], [79], [81].

Peaking birth rates during the dry season [9], [54], [72], [79] suggest that conception, or possibly implantation, may be most frequent during a narrow time interval [78]. Conception may be influenced by female condition at the time of mating, and related to resource availability, yet is probably also under some form of genetic or clocklike control, as is often the case in mammalian reproduction [87]. In giraffes, gestation time is roughly 14–16 months (e.g., [88]–[91]). Whereas giraffes reportedly mate throughout the year, based on the limited data available on births, the bulk of conceptions probably occur late in the wet season [78]. This may also be the time of optimal female condition due to the abundance of browse during the preceding period [78], [92], allowing reproductive females to recover from the energy deficit generated during gestation and lactation. Thus, reproductive timing may provide benefits with regard to parturition and subsequent weaning, but also with respect to conception, and maximizes the condition of both female and offspring. This notion is supported by the fact that giraffe calf recruitment is positively correlated with late dry season precipitation (i.e. earlier than normal green-up) over the preceding five-year period [93]. Similar situations seem to occur in elephants [94] and African buffalo [95], where conception is tightly linked to higher levels of NDVI – a measure of vegetation greenness indicating the availability of browse (Table 1).

An alternative mechanism that might explain the relation between genetic divergence and seasonal timing of rainfall could involve seasonal variation in habitat use as a response to differences in the timing of maximum rainfall and the associated availability of browse. Resource tracking has clear selective advantages and is one of the likely underlying causes of migration on both small and large scales. Although giraffes are capable of travelling large distances [5], they often have small ranges and exhibit localized responses to seasonal variation in resource availability [54]. For instance, when rainfall peaks in one area, giraffes in that area may be able to travel large distances because of the widespread availability of browse. However, this will coincide with a dry period in the adjacent regions, where giraffes may be confined to small patches of habitat with sufficient resources. Such an effect could render populations geographically isolated. In addition, habitat preferences – which are suggested to be different among males and females [85] – may limit the effective ranges of individual giraffes or populations in a given season. Thus, small home ranges that track the availability of browse associated with local to regional differences in maximum rainfall could facilitate the isolation of giraffe populations through neutral evolutionary processes. Finally, exposure to specific rainfall regimes could increase a given individual's preference for those natal cues through natal habitat preference induction (NHPI; e.g., [96] and references therein). Dispersing individuals might preferentially disperse to areas with habitat characteristics similar to those in the natal habitat. Such a scenario is independent of selection, but relies on imprinting of habitat cues during early stages of development.

In our hypothesis of ecologically mediated maintenance of population divergence, differences in reproductive timing need not act alone. For instance, mate recognition mechanisms may also contribute to isolation. In this context, differences in pelage pattern may serve as visual cues in mate choice, possibly through imprinting on the conspecific pelage pattern during the early stages of life [7]. However, to our knowledge no field data exist on the use of pelage patterns in mate recognition. To better understand the detailed mechanisms of isolation in contact zones, further studies of mate choice and habitat use are needed.

## Conclusions

We have shown that among the predictive variables considered, regional differences in the seasonal timing of rainfall and the associated timing of green-up best discriminate among the three East African giraffe taxa, and that general habitat differences, dispersal barriers, and geographic distance do so less effectively. One explanation for this striking relation may be related to reproductive asynchrony, suggesting regional adaptation of the reproductive cycle to the differential timing of green-up. This scenario might represent a form of ecologically-mediated reproductive isolation consistent with a growing body of work that suggests that selection can produce or maintain the divergence between ecologically distinct groups [2], [97]. Theory shows the efficacy of differential timing of the seasons or phenology on driving reproductive isolation of parapatric and sympatric populations [98], and studies of natural systems suggest that selection on timing of host plant flowering can lead to sympatric or micro-allopatric speciation of insects [19]. Similarly, our results might be explained by selection associated with timing of annual events, facilitating the maintenance of genetic and phenotypic divergence on regional scales in large, highly mobile animals. However, alternative mechanisms are also plausible. These might be related to differences in seasonal timing of rainfall, such as resource tracking and resulting seasonal allopatry, or to other factors, such as mate recognition based on pelage patterns. We have described a striking correlation between spatially divergent timing of maximum rainfall and giraffe divergence, warranting further research to better understand the exact nature of the relation and its potential role in maintaining giraffe population divergence.

## Supporting Information

Figure S1 **Response curves for variables entered in generalized dissimilarity models that were selected in the model as important in explaining the observed variation.** The maximum value of each variable is indicative of its importance in the model. Response curves are shown for models of Nei's D (A, B) and Fst (C, D) genetic distances with a set of predictor variables consisting of monthly precipitation, geographic distance, and a cost distance based on either altitude and ruggedness (A, C) or human population density (B, D).
(TIF)

Figure S2 **Monthly calving frequencies of giraffe in the Serengeti (solid line) and Nairobi National Parks (broken line).** Adapted from [54].
(TIF)
