# Supplementary figures and images for: Regional Differences in Seasonal Timing of Rainfall Discriminate between Genetically Distinct East African Giraffe Taxa
Source: PLoS One. 2013 Oct 23;8(10):e77191. doi: 10.1371/journal.pone.0077191 (PMC3806738; doi:10.1371/journal.pone.0077191)

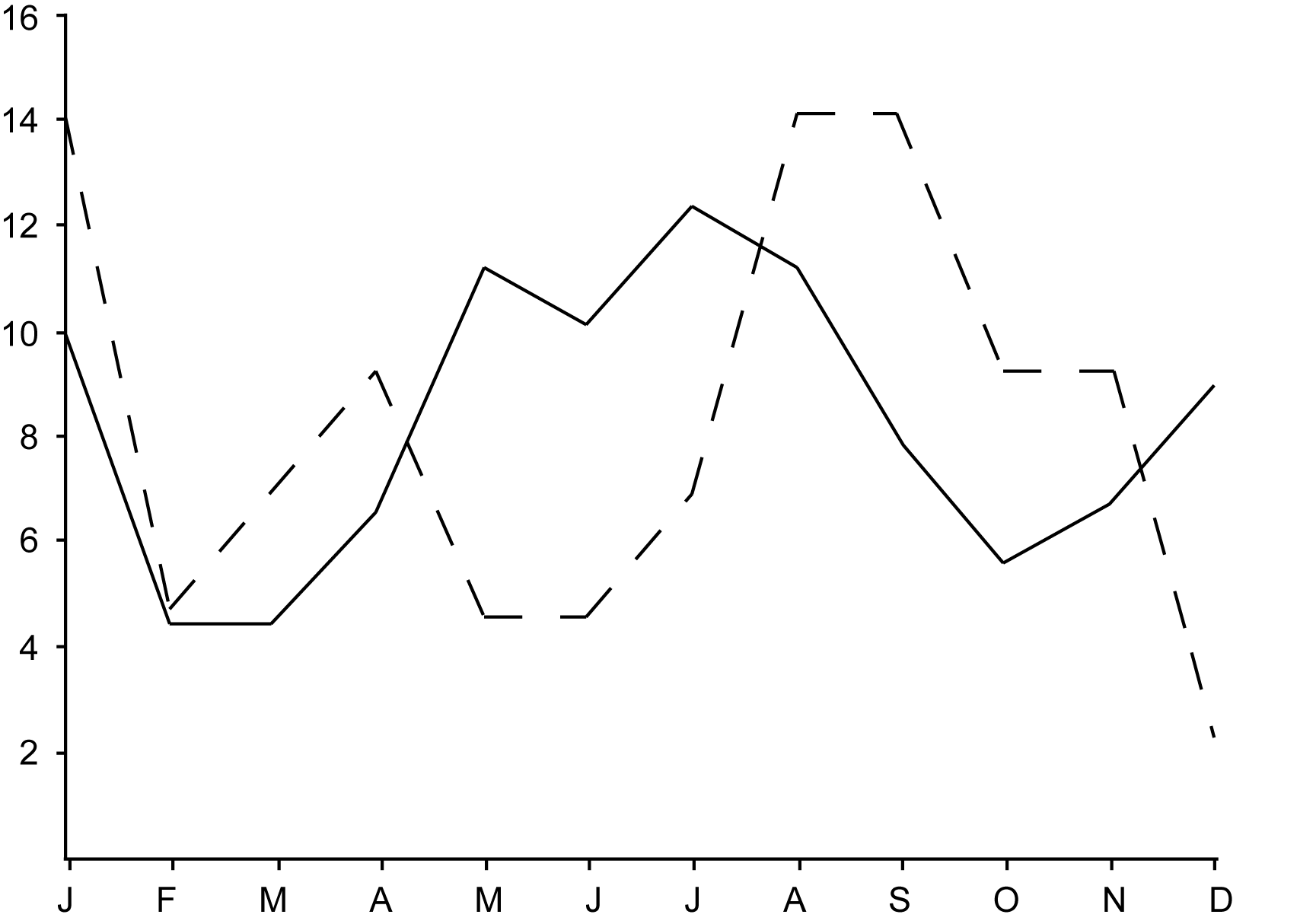

Supplement: Figure S2 — Monthly calving frequencies of giraffe in the Serengeti (solid line) and Nairobi National Parks (broken line). Adapted from [54]. (TIF) [file pone.0077191.s002.tif]
